# Proteomic alterations of HDL in youth with type 1 diabetes and their associations with glycemic control: a case–control study

**DOI:** 10.1186/s12933-019-0846-9

**Published:** 2019-03-28

**Authors:** Evgenia Gourgari, Junfeng Ma, Martin P. Playford, Nehal N. Mehta, Radoslav Goldman, Alan T. Remaley, Scott M. Gordon

**Affiliations:** 1 Division of Pediatric Endocrinology, Department of Pediatrics, Georgetown University, Washington, DC 20016 USA; 2 Proteomics and Metabolomics Shared Resource, Georgetown University Medical Center, Washington, DC USA; 3 Department of Oncology, Lombardi Comprehensive Cancer Center, Georgetown University Medical Center, Washington, DC USA; 4 Section of Inflammation and Cardiometabolic Diseases, National Heart, Lung, and Blood Institute, National Institutes of Health, Bethesda, MD USA; 5 Lipoprotein Metabolism Section, National Heart, Lung and Blood Institute, National Institutes of Health, Bethesda, MD USA; 6 Saha Cardiovascular Research Center, University of Kentucky, Lexington, KY USA; 7 Department of Physiology, University of Kentucky, Lexington, KY USA

**Keywords:** Proteomics, HDL, Type 1 diabetes, A1BG, ITIH4, Cardiovascular

## Abstract

**Background:**

Patients with type 1 diabetes (T1DM) typically have normal or even elevated plasma high density lipoprotein (HDL) cholesterol concentrations; however, HDL protein composition can be altered without a change in cholesterol content. Alteration of the HDL proteome can result in dysfunctional HDL particles with reduced ability to protect against cardiovascular disease (CVD). The objective of this study was to compare the HDL proteomes of youth with T1DM and healthy controls (HC) and to evaluate the influence of glycemic control on HDL protein composition.

**Methods:**

This was a cross-sectional case–control study. Blood samples were obtained from patients with T1DM and HC. HDL was isolated from plasma by size-exclusion chromatography and further purified using a lipid binding resin. The HDL proteome was analyzed by mass spectrometry using label-free SWATH peptide quantification.

**Results:**

Samples from 26 patients with T1DM and 13 HC were analyzed and 78 HDL-bound proteins were measured. Youth with T1DM had significantly increased amounts of complement factor H related protein 2 (FHR2; adjusted P < 0.05), compared to HC. When patients were analyzed based on glucose control, several trends emerged. Some proteins were altered in T1DM and not influenced by glycemic control (e.g. FHR2) while others were partially or completely corrected with optimal glucose control (e.g. alpha-1-beta glycoprotein, A1BG). In a subgroup of poorly controlled T1DM patients, inter alpha trypsin inhibitor 4 (ITIH4) was dramatically elevated (P < 0.0001) and this was partially reversed in patients with optimal glucose control. Some proteins including complement component C3 (CO3) and albumin (ALB) were significantly different only in T1DM patients with optimal glucose control, suggesting a possible effect of exogenous insulin.

**Conclusions:**

Youth with T1DM have proteomic alterations of their HDL compared to HC, despite similar concentration of HDL cholesterol. The influence of these compositional changes on HDL function are not yet known. Future efforts should focus on investigating the role of these HDL associated proteins in regard to HDL function and their role in CVD risk in patients with T1DM.

*Trial registration* NCT02275091

**Electronic supplementary material:**

The online version of this article (10.1186/s12933-019-0846-9) contains supplementary material, which is available to authorized users.

## Introduction

Patients with type 1 diabetes (T1DM) are at increased risk for cardiovascular disease [1]. This risk starts early in life as there is evidence of subclinical cardiovascular disease (CVD) in youth with T1DM [2]. The primary risk factors that have been attributed to CVD in this population include poor glycemic control, hypertension, obesity, and dyslipidemia [1, 3]. Dyslipidemia, characterized by non-HDL cholesterol > 130 mg/dL was found in 27.7% and low HDL < 35 mg/dL in only 3.4% of a large cohort of 682 children with T1DM [4]. In the SEARCH study, among 512 youth with T1DM and 188 healthy controls, the prevalence of low HDL < 35 mg/dL was 10.3%, 7.6% and 5% among healthy children and youth with T1DM and optimal or suboptimal control, respectively [5]. In general, low HDL levels in patients with T1DM is not as frequent as other types of dyslipidemia and the HDL profile can often even be favorable in patients with T1DM. However, despite the normal HDL cholesterol, the function of HDL can be impaired in some patients with T1DM [6–8]. The primary cardioprotective functions of HDL include the following: (i) prevention of the oxidation of LDL (ii) cholesterol efflux from the vessel wall and transport of cholesterol to the liver and (iii) anti-inflammatory function [9].

Alterations in the protein composition of HDL can affect its protective functions [7, 10]. For example, adults with T1DM and low levels of the protein apolipoprotein A-I (apoA-I) on the HDL particles were more likely to develop CVD [7]. Adult patients with T1DM and subclinical atherosclerosis have decreased levels of the potent antioxidant protein paraoxonase-3 (PON3) and PON3 concentration correlates with the anti-inflammatory function of HDL [9]. HDL proteome studies done in patients with type 2 diabetes (T2DM) have found alterations of HDL proteins linked to increased CVD risk [11, 12].

The influence of T1DM on the HDL proteome in youth with T1DM has not been reported. Our goal was to test the hypothesis that youth with T1DM have protein alterations on HDL that could play a role in modifying HDL function and contribute to an increased risk for CVD. In this study, SWATH mass spectrometry was used for label-free relative quantification of the HDL proteome. The protein composition of HDL was compared between youth with T1DM and healthy controls (HC) and the effect of glycemic control on HDL bound proteins was evaluated.

## Methods

### Study participants

All participants were enrolled in the clinical protocol “Identifying children with type 1 diabetes at high risk for CVD” (Clinical Trials Number: NCT02275091) that was approved by the Georgetown-Howard Universities Center for Clinical and Translational Science Institutional Review Board (IRB). Written informed consent was obtained from all parents of pediatric patients and all adult patients and assent was obtained from all children < 18 years old. Youth with T1DM and healthy controls between the ages of 12 and 21 years were eligible for participation. Subjects on lipid lowering medications were excluded from participation.

Recruitment of study participants was done by sending IRB approved letters to the patients with T1DM who are followed at the Pediatric Endocrine Divisions of Georgetown University (GU) and Children’s National Health System in Washington, DC. Healthy controls were recruited mainly by sending letters to families of children who had their well-child visits in the pediatric clinic of GU.

A research nurse conducted the anthropometric measurements and the BMI z-score was calculated using the CDC charts. Lipoprotein concentrations were measured using Liposcience NMR spectroscopy at the National Institutes of Health Clinical Center as previously described [13].

### Proteomics experiments

Preparation of samples for proteomics experiments was done following a number of steps as previously described [14, 15] and briefly summarized below: (1) purification of HDL from serum by size-exclusion chromatography; (2) pooling of all HDL containing fractions; (3) application of pooled HDL to lipid binding resin; (4) washing of HDL on resin to remove contaminating proteins; (5) trypsin digest of HDL on resin (overnight); (6) washing of the resin to collect tryptic peptides; (7) reduction and carbamidomethylation (DTT and iodoacetamide, respectively); and (8) sample desalting using ZipTips. The samples were dried down for proteomics analysis by using a nanoAcquity UPLC coupled with a TripleTOF 6600 mass spectrometer. First, we established a protein library of all HDL proteins detectable by mass-spectrometry (MS) for the analysis of a sample pool from all the subjects (with equal amount of proteins combined). The pooled sample was analyzed with 4 consecutive runs data in data dependent acquisition (DDA) mode. We then ran each sample individually via label-free SWATH data independent acquisition (DIA) to quantify each of these proteins in the samples. Specifically, peptides in each sample were dissolved into 20 µL of 0.1% formic acid. For spectra library generation, the pooled sample was loaded onto a C18 Trap column (Waters Acquity UPLC Symmetry C18 NanoAcquity 10 K 2G V/M, 100 A, 5 μm, 180 μm × 20 mm) at 15 µL/min for 4 min. Peptides were then separated with an analytical column (Waters Acquity UPLC M-Class, peptide BEH C18 column, 300 A, 1.7 μm, 75 μm × 150 mm) which was temperature controlled at 40 °C. The flow rate was set as 400 nL/min. A 60-min gradient of buffer A (2% ACN, 0.1% formic acid) and buffer B (0.1% formic acid in ACN) was used for separation: 1% buffer B at 0 min, 5% buffer B at 1 min, 45% buffer B at 35 min, 99% buffer B at 37 min, 99% buffer B at 40 min. The gradient went back to 1% buffer B to equilibrate the column for 20 min. The TripleTOF 6600 mass spectrometer was operated with an ion spray voltage of 2.3 kV, GS1 5 psi, GS2 0, CUR 30 psi and an interface heater temperature of 150 °C. The mass spectra were recorded with Analyst TF 1.7 software in the data dependent acquisitions (DDA) mode. Each cycle consisted of a full scan (m/z 400–1800) and fifty (IDAs) (m/z 100–1800) in the high sensitivity mode with a 2+ to 5+ charge state. Rolling collision energy was used. For SWATH acquisition, each of the samples was injected individually into the same NanoUPLC-MS/MS system (same settings as above) but acquired by repeatedly cycling through 32 consecutive 25-Da precursor isolation windows, generating time-resolved fragment ion spectra for all the analytes detectable within the 400–1200 m/z precursor range.

### Proteomics data analysis

For spectra library generation, raw mass spectra files after DDA acquisition of the pooled sample were submitted for combined searches using Protein Pilot version 5.0 software (Sciex) utilizing the Paragon and Progroup algorithms [16] and the integrated false discovery rate (FDR) analysis function [17]. MS/MS data were searched against the NCBI Homo Sapiens of the Uniprot-Sprot database containing 20,316 entries (downloaded on June 2, 2015). Trypsin was selected as the enzyme and carbamidomethylation was set as a fixed modification on cysteine. Variable peptide modifications included only methionine (M) oxidation. Other search parameters included instrument (TripleTOF 6600), ID Focus (Biological modifications), search effort (Thorough), false discovery rate (FDR) analysis (Yes), and user modified parameter files (No). The proteins were inferred based on the ProGroupTM algorithm associated with the ProteinPilot software. The detected protein threshold in the software was set to the value which corresponded to 1% FDR. Peptides were defined as redundant if they had identical cleavage site(s), amino acid sequence, and modification.

For the label-free SWATH quantification, data from each sample was pre-processed by PeakView 2.1 (Sciex), with the default settings: (1) peptide filter: # of peptides per protein: 6; # of transitions per peptide: 6; peptide confidence threshold: 99%; FDR threshold: 1%; (2) extracted ion chromatogram (XIC) Options: XIC extraction window (min): 5; XIC width (ppm): 75. Retention time was calibrated by selecting 6 peptides with retention time across the whole HPLC gradient. Peak of each transition produced was then manually checked and curated, with only transitions detected in all samples and showing signal to noise (S/N) > 10 chosen for peak area calculation. The peptide response was calculated as the sum of all ion intensity for all curated transitions. The sum of response of all curated peptides in each protein was used for protein level quantification (Additional file 1: Table S1). The intensity of proteins was then normalized to the total ion intensity of each sample, with the ratio of protein representing the protein level in each sample (Additional file 2: Table S2).

### Cholesterol efflux analysis

To determine whether any of the proteins of interest are related to changes in the cholesterol efflux function of HDL, we measured HDL efflux capacity as previously described by our group [8]. In summary, HDL cholesterol efflux capacity assays were performed using the murine macrophage cell line, J774, based on published methods, [18–20]. Briefly, 3 × 10^5^ J774 cells/well were plated and radiolabeled with 2 µCi of ^3^H-cholesterol/mL for 24 h. ATP-binding cassette transporter A1 (ABCA1) was up-regulated by means of a 16-h incubation with 0.3 mmol/L 8-(4-chlorophenylthio)-cAMP. ApoB-depleted plasma (2.8%) was added to the efflux medium for 4 h. Liquid scintillation counting was added to quantify the efflux of radioactive cholesterol from the cells. Efflux was calculated using the following formula: (µCi of ^3^H-cholesterol in media containing 2.8% apoB-depleted subject plasma-µCi of ^3^H-cholesterol in plasma-free media/µCi of ^3^H-cholesterol in media containing 2.8% apoB-depleted pooled control plasma-µCi of ^3^H-cholesterol in pooled control plasma-free media). The pooled plasma was obtained from five healthy adult volunteers. All assays were performed in duplicates.

### Statistical analysis

Ion intensity data for each protein was tested for normality using the Shapiro–Wilk test (SigmaPlot 13.0). All protein ion intensity measurements were log_10_ transformed prior to analysis. To identify proteins with differential abundance between healthy control and T1DM groups we used two approaches: (A) Student’s t test was used on individual proteins, without correction for multiple comparisons. In proteomics data sets, common methods of adjustment for multiple comparisons testing often result in a high occurrence of false negatives [21]. To reduce the risk of omitting true positive hits from this analysis, these results were initially screened using more relaxed statistical criteria with a P value threshold of 0.05 without corrections for multiple comparisons. (B) To evaluate the data with consideration for multiple testing correction, we used the two stage step up false discovery rate method of Benjamini, Krieger and Yekutieli with a Q value cutoff of 5%. The ion intensity dataset included one missing data point (out of 3042 observations). The missing data point was ignored in subsequent calculations. Fold-change was calculated as the ion intensity in T1DM divided by the same protein’s intensity in the healthy control group. Linear regression and ANOVA analyses were performed under described parameters using GraphPad Prism 7 software.

## Results

### Study population

We analyzed the HDL proteome in 26 patients with T1DM and 13 HC (Table 1). Patients were matched for age, sex, BMI, and clinical lipid measures. The average duration of diabetes was 2.9 ± 1.1 years in the T1DM group and the average HbA1c was 8.9 ± 1.8% in the T1DM and 5.3 ± 0.3% in the HC, P ≤ 0.001.

**Table 1:** Clinical characteristics of heavy control and T1DM patients

	Healthy controls	Type 1 diabetes	P value
n	13	26	–
Age (years)	16.8 ± 1.4	16.9 ± 1.9	0.99
Female sex, n (%)	11 (84%)	18 (69%)	–
BMI z score	0.1 ± 1.0	0.4 ± 0.1	0.79
HbA1c %	5.3 ± 0.3	8.9 ± 1.8	< 0.0001
Diabetes duration	–	2.9 ± 1.1	–
hsCRP	0.33 ± 0.2	1.9 ± 3.5	0.60
HDL cholesterol (mg/dL)	59.0 ± 12.0	59.7 ± 8.6	0.99
LDL cholesterol (mg/dL)	60.5 ± 28.8	72.6 ± 21.9	0.60
Triglyceride (mg/dL)	81.6 ± 34.5	81.7 ± 36.3	0.99

P values are derived from t tests with Holm–Sidak correction for multiple comparisons

### HDL proteome analysis

We developed a library for MS-based label-free quantitation of HDL-bound proteins, using a subset of samples from each study group. This approach quantified 78 proteins that are present in HDL fractions in both T1DM and HC (matched with 229 peptides and 1142 transitions; > 95% confidence) after manual curation (i.e., only transitions showing S/N > 10 were selected for quantification) (Additional file 1: Table S1).

**Fig. 1:**
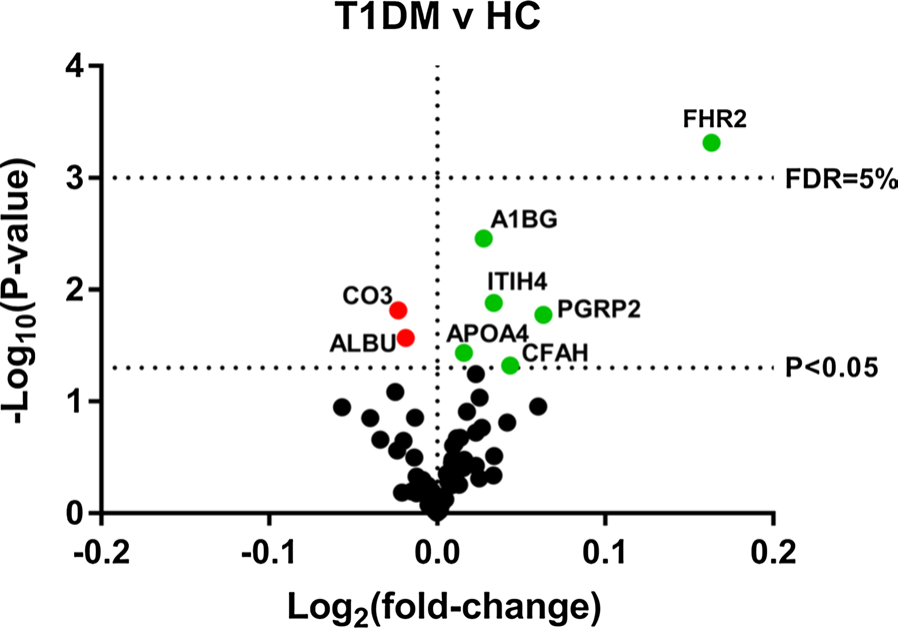
The HDL proteome is altered in youth with type 1 diabetes. HDL was isolated from youth with T1DM and healthy controls then proteome composition was analyzed by mass spectrometry to quantify 78 HDL-bound proteins. This volcano plot displays the differences in protein abundance detected between the healthy controls (HC) and T1DM subjects. Each point represents one of the 78 detected proteins. Green points are proteins with positive fold change (i.e. increase in T1DM) and red points are negative fold changes. Horizontal dotted lines indicate statistical thresholds for a false discovery rate (FDR) of 5% by the method of Benjamini, Krieger and Yekutieli or a P-value < 0.05 calculated using two-tailed t-test without adjustment for multiple comparisons. *A1BG* alpha-1B-glycoprotein, *APOA4* apolipoprotein A-IV, *FHR2* factor-H related protein 2, *ITIH4* inter-alpha trypsin inhibitor 4, *PGRP2* peptidoglycan recognition protein 2, *ALBU* albumin, *CO3* complement C3, *CFAH* complement factor H

Analysis of the HDL proteome in all participants using an unadjusted t-test revealed 8 proteins that were potentially altered among the patients with T1DM and HC. Six of these proteins were elevated in HDL of youth with T1DM and two of them were decreased (Fig. 1). Youth with T1DM had significantly higher protein levels of alpha-1B-glycoprotein (A1BG; P = 0.003), apolipoprotein A-IV (APOA4; P = 0.037), complement factor H (CFAH; P = 0.0488), factor H related protein 2 (FHR2; P = 0.0005), inter-alpha trypsin inhibitor 4 (ITIH4; P = 0.013), peptidoglycan recognition protein 2 (PGRP2; P = 0.017) and lower levels of albumin (ALBU; P = 0.027) and complement C3 (CO3; P = 0.015) compared to HC. When these results were analyzed with more strict statistical approach by applying a false discovery rate threshold of 5%, only one protein was identified as statistically significant, FHR2 (P = 0.039).

**Fig. 2:**
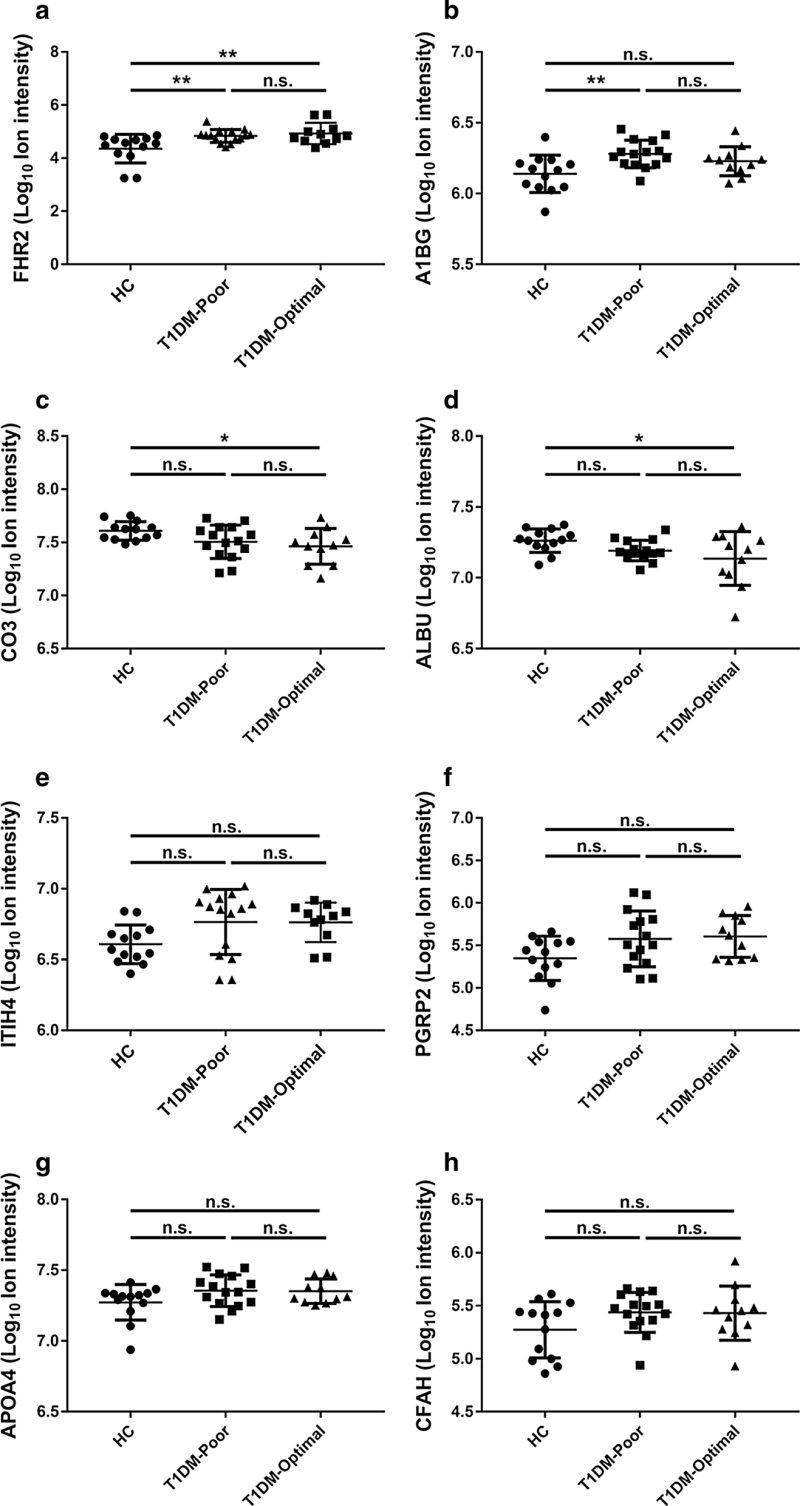
Effects of glycemic control on HDL-bound proteins. T1DM participants were separated into those with either “poor” or “optimal” glycemic control and levels of HDL-bound proteins detected by mass spectrometry were compared with healthy controls (HC) by one-way ANOVA with Dunnett’s correction for multiple comparisons. **a** *FHR2* factor-H related protein 2;** b** *A1BG* alpha-1B-glycoprotein;** c** *CO3* complement C3;** d** *ALBU* albumin;** e** *ITIH4* inter-alpha trypsin inhibitor 4;** f** *PGRP2* peptidoglycan recognition protein 2;** g** *APOA4* apolipoprotein A-IV;** h** *CFAH* complement factor H  *P < 0.05; **P < 0.01; n.s. indicates not statistically significant

**Fig. 3:**
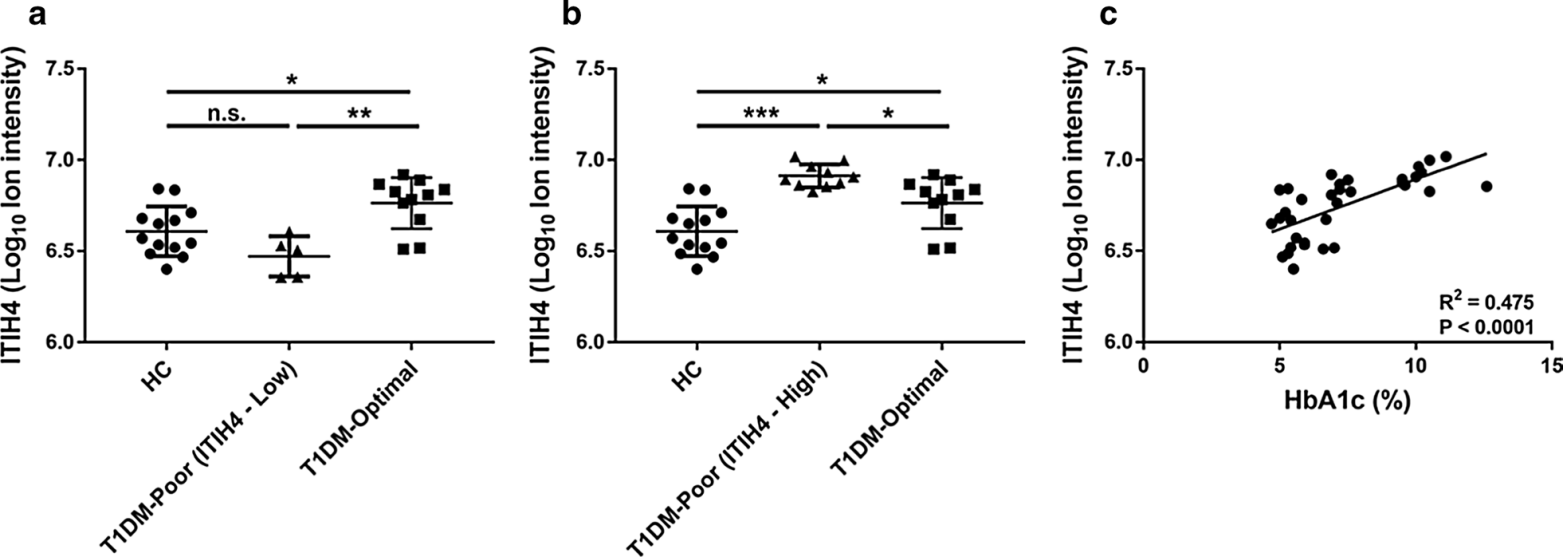
ITIH4 displays a unique bimodal distribution in T1DM subjects with poor glycemic control. **a**, **b** T1DM subjects with poor glycemic control were separated into ITIH4-Low and ITIH4-High groups and compared separately with the other groups by one-way ANOVA with Dunnett’s correction for multiple comparisons. **c** Linear regression analysis of entire cohort with T1DM poor (ITIH4-Low) subjects removed. *P < 0.05; **P < 0.01; ***P < 0.001; n.s. indicates not statistically significant. *ITIH4* inter-alpha trypsin inhibitor 4

To determine if HDL-bound proteins correlate with glycemic control, linear regression analysis was used to evaluate the relationships between measured proteins and HbA1c. Of those proteins potentially altered by type I diabetes status, A1BG (r^2^ = 0.23, adjusted P = 0.0152) was significantly associated with HbA1c (Table 2). To examine the effect of glycemic control in patients, we divided the T1DM cohort into poorly controlled (HbA1c > 7.6%, n = 15) and well-controlled (HbA1c ≤ 7.6%, n = 11) subjects. Comparison of proteins levels among the three subgroups revealed four different trends: (1) the protein is elevated in patients with T1DM and not affected by glycemic control (FHR2; Fig. 2a). (2) The protein is elevated in T1DM and partially returns to normal with glycemic control (A1BG; Fig. 2b). (3) The protein only differs in patients with well-controlled T1DM, suggesting possible effect of exogenous insulin (CO3 and ALBU; Fig. 2c, d). (4) The proteins are not affected by T1DM control status (ITIH4, PGRP2, APOA4, CFAH; Fig. 2e–h). A bimodal distribution of ITIH4 was apparent in subjects with poorly controlled T1DM (Fig. 2e). To further examine this effect, we isolated these two subpopulations and reanalyzed the relationship among the three groups. ITIH4 in subjects with poorly controlled T1DM and low ITIH4 was not different compared to HC (Fig. 3a). However, the poorly controlled T1DM subjects with high ITIH4 had significantly greater ITIH4 than HC and well-controlled T1DM subjects (Fig. 3b). Linear regression analysis of the cohort with the poorly controlled low ITIH4 subjects removed revealed a striking correlation between HDL-bound ITIH4 and HbA1c (Fig. 3c). Patient data including sex, BMI, hsCRP, or duration of diabetes did not correlate with ITIH4 high/low status.

**Table 2:** Linear regression of HbA1c with HDL-bound proteins

Protein	Slope	R square	P value	Adjusted P
A1BG	0.026 ± 0.008	0.23	0.0019	0.015
FHR2	0.076 ± 0.031	0.14	0.020	0.16
PGRP2	0.039 ± 0.021	0.09	0.064	0.51
ITIH4	0.024 ± 0.013	0.09	0.069	0.55
CFAH	0.031 ± 0.017	0.08	0.074	0.59
APOA4	0.013 ± 0.008	0.07	0.10	0.81
CO3	− 0.014 ± 0.011	0.04	0.20	1.60
ALBU	− 0.008 ± 0.009	0.02	0.41	3.26

P values adjusted for multiple comparisons by Bonferroni method

**Fig. 4:**
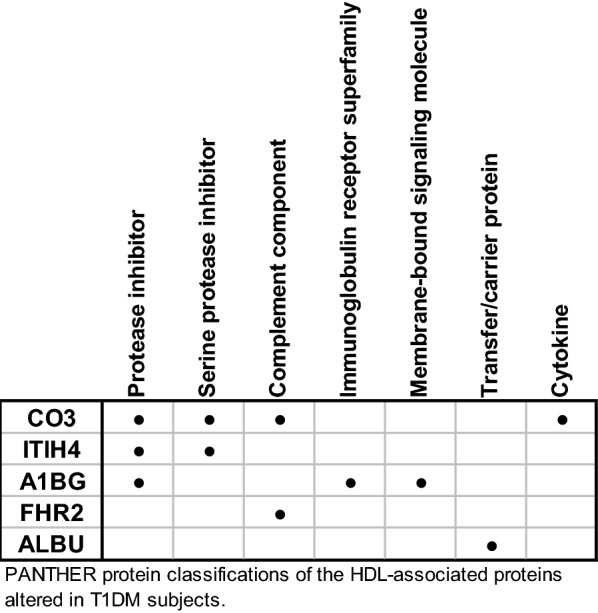
Functional classifications of HDL-associated proteins affected by T1DM. PANTHER protein classification system was used to identify known functions of each of the proteins altered by T1DM in this study. *CO3* complement C3, *ITIH4* inter-alpha trypsin inhibitor 4, *A1BG* alpha-1B-glycoprotein, *FHR2* factor-H related protein 2, *ALBU* albumin

Overall, five proteins were determined to be influenced by T1DM and glycemic control: CO3, ITIH4, A1BG, FHR2, and ALBU. These proteins were annotated with known functions using PANTHER protein classifications (version 14.0) (Fig. 4) [22]. The most represented protein classes are protease inhibitor (3/5), serine protease inhibitor (2/5), and complement component (2/5). Function enrichment analysis was performed using the PANTHER overrepresentation analysis with Fisher’s Exact test and FDR correction [23]. This analysis compares the frequency of protein class assignments within a list of proteins to the frequency of those function classes in the entire human proteome. The HDL-associated proteins altered by T1DM contains a significant enrichment of protease inhibitor (P = 0.0076, FDR adjusted) and serine protease inhibitor (P = 0.023, FDR adjusted).

### Cholesterol efflux analysis

As an indicator of HDL function, cholesterol efflux capacity (CEC) to apoB depleted plasma was measured. No differences in CEC were detected between the HC and T1DM groups (Additional file 3: Figure S1). Additionally, proteins influenced by T1DM status did not correlate with CEC.

## Discussion

This report describes, for the first time, proteomic alterations of HDL in a cohort of young adults with T1DM. We have identified several HDL-bound proteins that are significantly affected in patients with T1DM. We observed three clear trends in those affected proteins. FHR2 was increased in T1DM and not corrected by glucose control. This may suggest that this is caused by some consequence of the underlying condition and not circulating glucose levels. A1BG and ITIH4 were increased in T1DM and partially corrected by glucose control. Finally, CO3 and ALB are decreased in T1DM patients with well-controlled glucose. This may suggest the administration of insulin is influencing the association of these proteins with HDL.

Gene ontology analysis using PANTHER protein classifications revealed a significant enrichment of proteins with protease inhibitor function, particularly serine protease inhibitors. This indicates that the effect of T1DM on the HDL proteome may specifically influence the protease regulator activity of HDL. Protease regulator activity on HDL has been previously suggested to play an important role in atherosclerotic cardiovascular disease [24]. The following paragraphs discuss some potential roles for each of the affected proteins in HDL function and CVD risk.

FHR2, is a complement factor H (CFAH) related protein that resembles structurally and immunologically the complement factor H. While the complement pathway is a critical component of innate immunity, inappropriate complement activation has been linked to inflammation, diabetes, insulin resistance, atherosclerosis, and cardiometabolic diseases [25, 26]. Complement factor H is an important regulatory protein and prevents tissue damage from inappropriate complement activation [27]. Recently, it was shown that factor H binds to ApoE on HDL and this plays a role in regulating complement activation [28]. Furthermore, CFAH presence has also been linked with decreased insulin production from rat pancreatic cells and it may play a role in the pathogenesis of diabetes [27]. Whether the increased FHR2 in HDL of T1DM patients is related to the pathogenesis of T1DM remains to be explored. FHR2 has been previously detected on lipoproteins and it is thought to facilitate the adhesive response of neutrophils to lipopolysaccharides [27]. It is also possible that complement factor H and complement factor H related proteins (such as FHR2) play a role in lipid transport and regulate lipid homeostasis [29]. The mechanism by which CFAH and FHR2 interact to regulate HDL function in diabetes requires further investigation.

A1BG, alpha-1-B glycoprotein, belongs to the immunoglobulin family and its function is largely unknown [30, 31]. A1BG was found to be overexpressed in tissues of pancreatic ductal adenocarcinoma and liver cancer cell lines [30], while it was not detected in normal pancreatic tissue and hence could be useful as a tumor marker. A1BG was also reported to be elevated in the urine of normoalbuminuric patients with T1DM when compared to healthy controls; however, the urine levels of this protein did not correlate with HbA1c or urine microalbumin [31]. Whether this protein could serve as a urine biomarker of early diabetic kidney disease remains to be examined. Interestingly, pharmacogenomic studies have shown that polymorphisms in the *A1BG* gene can play a role in cardiovascular outcomes of patients treated with antihypertensive medications [32].

ITIH4, inter-alpha-trypsin inhibitor heavy chain H4, belongs to the liver-restricted serine protease inhibitor family. It is highly expressed in liver development and low levels have been found in hepatocellular and ovarian cancer [33, 34]. ITIH4 is elevated in urine of patients with T2DM and microalbuminuria and could possibly serve as a biomarker for diabetic kidney disease [35]. ITIH4 was also found to be downregulated after a very low caloric diet in patients with T2DM, possibly representing a biomarker of metabolic improvement [36].

We were unable to identify a clinical basis for the bimodal distribution of ITIH4 in the poorly controlled T1DM cohort. Using logistic regression analysis, high vs low ITIH4 grouping does not appear to correlate with age, sex, BMI, hsCRP, or duration of diabetes. However, the high ITIH4 group, which made up 66% of the poorly controlled cohort displayed a robust elevation compared to HC that, similar to A1BG, was partially corrected with glycemic control. These results suggest that better glycemic control could reverse the changes associated with overexpression of A1BG and ITIH4 on HDL. The fact that some protein alterations can be corrected by glycemic control and others cannot indicates that these HDL proteome effects may be mediated by different mechanistic pathways. Future studies could examine how glycemic control alters HDL structure and function and explore novel mechanisms to improve CVD risk in T1DM.

ALBU, albumin, is the most abundant protein in the plasma and binds to electrolytes, hormones, fatty acids and drugs. Relatively low serum albumin has also been used as a marker of increased mortality from CVD [37, 38]. Serum albumin has been found to be positively correlated with HDL and total cholesterol, and it is possible that low serum albumin reflects abnormalities in lipid metabolism and function [39]. Interestingly, glycated albumin was shown to decrease the anti-inflammatory function of HDL and impair the reversed cholesterol transport function, contributing to the development of CVD in patients with diabetes [40]. Whether the low albumin on HDL we found in our patients with T1DM reflects an imbalance between glycosylated and non-glycosylated forms of albumin requires further study.

CO3, complement factor 3, is produced by macrophages and plays a key initiating role in the activation of complement on the vascular endothelium, which triggers an inflammatory response, creating a vessel wall that is prone to atherosclerosis and increasing CVD risk [26]. CO3 has been previously detected on HDL [10, 41]. Vaisar et al. reported multiple complement regulatory proteins on HDL fractions [41]. Interestingly, in their study, subjects with cardiovascular disease had significantly elevated levels of CO3 on HDL fractions [41]. Others have shown that increased CO3 on HDL of patients with CVD, psoriasis and rheumatoid arthritis is linked to decreased cholesterol efflux [10, 42, 43]. Additionally, HDL-bound CO3 has been correlated with increased non-calcified plaque burden in patients undergoing coronary CT angiography [44]. The current study found lower CO3 in T1DM compared to HC (although statistically significant only in the “well-controlled” glycemic cohort), which suggests a protective effect of insulin given the evidence in literature. This could represent a protective role of HDL against CVD in T1DM subjects during the early stages of disease.

Both ALBU and CO3 were significantly lower only in the group of T1DM with optimal control compared to HC, suggesting that perhaps higher doses of insulin may play a role in altering their values. Insulin can increase the transcapillary escape of albumin as well as the urinary excretion of albumin and whether the lower albumin and CO3 on HDL could be secondary to these mechanisms requires further investigation [45, 46].

Strengths of our study include that this is a well-characterized cohort of youth with T1DM, with optimal and suboptimal glycemic control, along with detailed HDL proteome data. The combination of two-step size-exclusion chromatography and lipid interaction based HDL purification and SWATH-MS provides sensitive and robust label-free proteomic quantitation for multiple clinical samples. Limitations include the small sample size and lack of full functional studies for each protein of interest. Our goal for this project was to provide an initial characterization of the proteomic differences in HDL composition between youth with T1DM and HC and identify associations with glycemic control. Future studies will further investigate the functional roles of the identified proteins that differed on HDL from T1DM patients.

Our group has previously published differences in the cholesterol efflux values between T1DM and HC in a larger cohort [8], but in this particular subset of participants we did not detect differences in cholesterol efflux, potentially because of the smaller sample size, the well-controlled status in half of the T1DM participants and their short diabetes duration. There were no significant correlations between the specific proteins of interest and cholesterol efflux. The lack of a group wide effect on HDL’s cholesterol efflux capacity, is not necessarily surprising considering that the HDL-cholesterol levels are not different in this cohort and the protein changes detected here do not include proteins known to influence efflux (e.g. apoA-I, serum amyloid A, etc.) [44, 47]. Based on our functional classification analysis, it seems likely the influence of T1DM on HDL may be more strongly tied to HDL’s roles in protease regulation and inflammation [24].

## Conclusions

In summary, we found altered protein composition of HDL in youth with T1DM compared to HC by combining size-exclusion chromatography-based HDL purification and SWATH-MS-based label-free proteomic quantitation. The specific proteomic changes suggest a possible connection with increased risk for cardiovascular disease, via multiple pathways relevant to known functions associated with the affected proteins. Future studies should examine the specific role of each protein on altering known functions of HDL such as anti-inflammatory activity, cholesterol efflux capacity, and prevention of oxidation of LDL or the exploration of novel HDL functions. Larger epidemiologic studies could also examine the association of the altered HDL proteins with cardiovascular events. Finally, the association of glycemic control with some of these HDL associated proteins suggests the possibility that better glycemic control could reverse compositional abnormalities of HDL and potentially decrease CVD risk by restoring HDL function.

## Additional files

**Additional file 1: Table S1** Table of ion transitions used for proteomics analysis.

**Additional file 1: Table S2** Raw ion intensity data from proteomics analysis.

**Additional file 3: Figure S1.** Cholesterol efflux capacity (CEC) in T1DM subjects and healthy controls.

## Abbreviations

A1BG: alpha-1B-glycoprotein
ALBU: albumin
APOA4: apolipoprotein A-IV
CEC: cholesterol efflux capacity
C03: complement C3
CFAH: complement factor H
CVD: cardiovasular disease
FHR2: factor H related protein 2
GU: Georgetown University
HC: healthy controls
HDL: high density lipoprotein
IRB: Institutional Review Board
ITIH4: inter-alpha trypsin inhibitor 4
PGRP2: peptidoglycan recognition protein 2
PON3: protein paraoxonase-3
T1DM: type 1 diabetes
T2DM: type 2 diabetes
