# Supplementary material for: Proteomic alterations of HDL in youth with type 1 diabetes and their associations with glycemic control: a case–control study
Source: Cardiovasc Diabetol. 2019 Mar 28;18:43. doi: 10.1186/s12933-019-0846-9 (PMC6437869; doi:10.1186/s12933-019-0846-9)
Supplement: Supplementary file 3 — Additional file 3: Figure S1. Cholesterol efflux capacity (CEC) in T1DM subjects and healthy controls. [file 12933_2019_846_MOESM3_ESM.docx]

**Figure S1. Cholesterol efflux capacity (CEC) in T1DM subjects and healthy controls.** Transfer (efflux) of radiolabeled free cholesterol from J774 macrophages to apoB-depleted plasma from study subjects. Data are normalized to a standard pool of healthy donors (CEC = 1). No statistically significant differences were detected among groups by one-way ANOVA with Tukey correction.
